# Hyperspectral Imaging Database of Human Facial Skin

**DOI:** 10.1177/00037028241279323

**Published:** 2024-09-24

**Authors:** Andreia E. Gomes, Sérgio M. C. Nascimento, João M. M. Linhares

**Affiliations:** Physics Center of Minho and Porto Universities (CF-UM-UP), 56059 University of Minho, Braga, Portugal

**Keywords:** Skin color, human faces, hyperspectral imaging, spectrophotometry, spectroradiometry, colorimetry, spectral reflectance

## Abstract

The perceived color of human skin is the result of the interaction of environmental lighting with the skin. Only by resorting to human skin spectral reflectance, it is possible to obtain physical outcomes of this interaction. The purpose of this work was to provide a cured and validated database of hyperspectral images of human faces, useful for several applications, such as psychophysics-based research, object recognition, and material modeling. The hyperspectral imaging data from 29 human faces with different skin tones and sexes, under constant lighting and controlled movements, were described and characterized. Each hyperspectral image, which comprised spectral reflectance of the whole face from 400 to 720 nm in 10 nm steps at each pixel, was analyzed between and within nine facial positions located at different areas of the face. Simultaneously, spectral measurements at the same nine facial positions using conventional local point and/or contact devices were used to ascertain the data. It was found that the spectral reflectance profile changed between skin tones, subjects, and facial locations. Important local variations of the spectral reflectance profile showed that extra care is needed when considering average values from conventional devices at the same area of measurement.

## Graphical abstract

**Graphical abstract:**
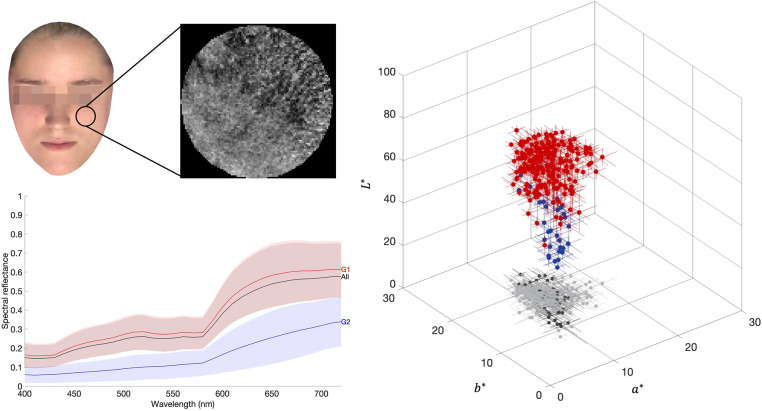
This is a visual representation of the abstract.

## Introduction

The color of human skin is important to the perception of several aspects of the face, e.g., beauty,^1–6^ overall health,^7–11^ emotional status, or psychological state.^5,7,8,10,11^ It is also important in social interactions and social status impressions.^12–16^

Skin is a complex structure with functional and physical variations across its surface and within its layers,^17,18^ greatly heterogeneous due to the presence of distinct elements such as freckles, marks, veins, rashes, or pigmentation. The perceived color of the skin is the result of optical interactions between light and skin physio-anatomical characteristics such as melanin, hemoglobin, and carotene.^17^ The properties of the human skin spectral reflectance are mainly influenced by hemoglobin and melanin concentrations and/or absorption.^19–23^ Consequently, these features are generally considered for skin color analysis.^24–27^ Skin color changes may be harder to detect in darker skin since these have lower rates of light reflection and higher melanin concentrations,^23,28,29^ so they should be considered differently.

The characterization of the physical properties of the human skin has been a subject of interest, mainly to achieve descriptive models capable of simulating the intricacies of the perception of skin under varying illumination for realistic rendering on digital media.^22,24,25,29–34^ The full characterization may also help under clinical conditions,^22^ as the color of the human skin may be used as an indicator for different medical diagnostic conditions,^31,32,35–38^ the development of skin treatments,^39,40^ and the development of cosmetic products to improve skin, such as makeup and skin care. It is also used for marketing purposes helping the selection of the best skin color to advertise a specific product and/or goal for each type of consumer.^18^ Nevertheless, the use of images of the skin to access its features may be hindered when the spectral properties are not considered, in particular, if data are used for artificial intelligence training or operations.^41^

Available methods to acquire spectral data on human skin are generally based on local point-contact spectrometers that are easy to handle from a practical point of view and provide precise and reproducible data.^23,26,42,43^ However, colorimetric analysis of the human skin using these devices is limited to small sampling areas, providing only local uncontextualized spatial information, and hindering the comparisons among different areas of measurements, successive measurements, or even local variations around a specific skin spot.^22,26,42–44^ For a global analysis where spatial-spectral and/or colorimetric variations are required, such spectral data do not provide meaningful information. Alternatively, hyperspectral imaging is a noncontact and noninvasive method that enables the recording of spectral components of the reflected light simultaneously with the corresponding spatial information.^22^ Existing databases of skin spectral reflectance may cover only the visible range of the electromagnetic spectrum,^29,33^ or provide spectral information over the near-infrared region of the electromagnetic spectrum.^45,46^

Conventional RGB devices (digital color cameras) can also be used to acquire the color information of the skin, but these devices only retrieve information from three broad regions of the visible light spectrum, namely short, medium, and large wavelength spectral regions. As these devices produce only approximate colors when spectral reflectance is reconstructed from these data^47,48^ a loss of information occurs. By opposition, hyperspectral imaging does not compress the spectral information into such broad bands, preserving the spectral details needed for proper representation of the human skin.^49–51^ It also has the capability of highlighting features of the captured image that can only be visible at specific wavelengths.^52^

The applications of hyperspectral imaging of the skin are diverse. In the representation and analysis of the color of the skin, the technique was used to analyze subtle color variations along the skin tissue,^28^ to model and render the appearance of human skin,^24,53–55^ spectral manipulation of lighting of the skin,^56^ skin's in vivo color changes^57^ due to the influence of hemoglobin concentration and/or oxygen saturation,^58–60^ skin disorders diagnosis and assessment,^61–66^ skin histological analysis by coupling a hyperspectral imaging system with a microscope,^50^ the treatment of skin disorders,^66–69^ and the dyes in tattoos,^70^ thus being considered as a useful tool for dermatological purposes.^71^

Regarding databases of spectral information on human skin, there is a limited amount of information.^46,56,72,73^ The few hyperspectral facial databases (HSFD) referenced in the literature, such as Carnegie Mellon University (CMU HSFD),^52,74,75^ Hong Kong Polytechnic University (PolyU-HSFD),^52,73,76^, Imaging, Robotics, and Intelligent Systems (IRIS) Laboratory from University of Tennessee (IRIS-M,^52,77–79^ and IRIS-HFD-2014^52,76,80^), University of Western Australia (UWA HSFD),^52,76^ Stanford University,^52,76,81^ and Lippman2000 (Munsell Color Science Laboratory, New York),^82^ are mainly targeted for facial recognition^52,76^ or skin modeling,^83,84^ and have some limitations, i.e., predominantly composed of light skin tones, a lack of movement control due to the absence of support for the head and/or body, eye movements due to blinking, or eye irritation due to photo lighting, necessary multiple sessions, illuminant uncontrolled variations, constant exposure time unadapted to the lighting conditions, reduced number of spectral bands, or low signal-to-noise ratios. If the skin of human faces without facial expressions is needed, more reliable information is required. The database proposed in this study might be useful for psychophysical and/or behavioral experiments,^2,12,85,86^ devices of neural network resources,^69^ automatized color classification,^87^ or skin modeling appearance.^25,88,89^

The main goal of this study was to introduce a carefully curated and characterized hyperspectral image database of human faces. Faces with different skin tones of male and female were imaged and strategically positioned to reduce facial movements and optimize lighting conditions. Data were acquired in the laboratory with specific care to reduce the presence of nonskin features (teeth, hair, eyes open, beard, make-up, and accessories). An analysis was conducted for both inter- and intra-group variations across different facial locations for each skin group. These measures were ascertained with spectral measurements made with conventional local points and/or contact. Analysis of the data revealed important local variations in the spectral reflectance profile that cannot be detected when using local point measuring devices.

## Experimental

### Materials and Methods

The ethical issues of the experimental protocol were approved by the Ethics Committee for Research in Life and Health Sciences of the University of Minho (CEICVS 052/2021).

### Participants

Participants with diverse skin color and type were recruited from the academic population from the University of Minho. Participants with any diagnosed and/or visible skin problems, anomalies, or facial hair were excluded from the image acquisition. Before the acquisition, the faces of the participants were thoroughly cleaned to remove any facial cream.

The color of the facial skin was classified using von Luschan's Chromatic Scale which comprises 36 physical-colored samples correlated with the spectral profile of human skin, making this scale suitable for the evaluation of the skin color,^90^ and Fitzpatrick's scale with six skin phototypes based on the skin behavior when exposed to the radiation of the sun.^91,92^

Twenty-nine human faces were measured and divided into two different groups based on skin color, namely Group 1 and Group 2. Group 1 includes skin color tones of the von Luschan scores from 1 to 15 and Fitzpatrick phototypes from I to III, whereas Group 2 includes the von Luschan scores from 16 to 36 and Fitzpatrick phototypes between IV and VI. Table I summarizes the distribution of the participants across the two groups, their sex, and their average age (Examples of the faces assigned to each group defined in Table I are presented in Figure S1 in the Supplemental Material).

**Table I.** Classification of the participants according to their scores in the Von Luschan scale, the Fitzpatrick phototype scale, and the corresponding number of participants, their sex, and their average age.

	Group 1	Group 2
Von Luschan score	7–11	25–32
Fitzpatrick phototype	II–III	V–VI
Number of faces	25	4
Sex (male/female)	3/22	0/4
Age (average ± standard deviation)	20.2 ± 1.5	18.8 ± 1.5

### Spectral Data Measurements

Spectral data were acquired using a telespectroradiometer (TSR), a contact spectrophotometer (SPM), and a hyperspectral imaging system (HIS). Data obtained were compared to the data available elsewhere using SPM^19–21,33,44,46,72,93,94^ and/or TSR.^29,95,96^

### Spectroradiometric Measurements

**Figure 1.**
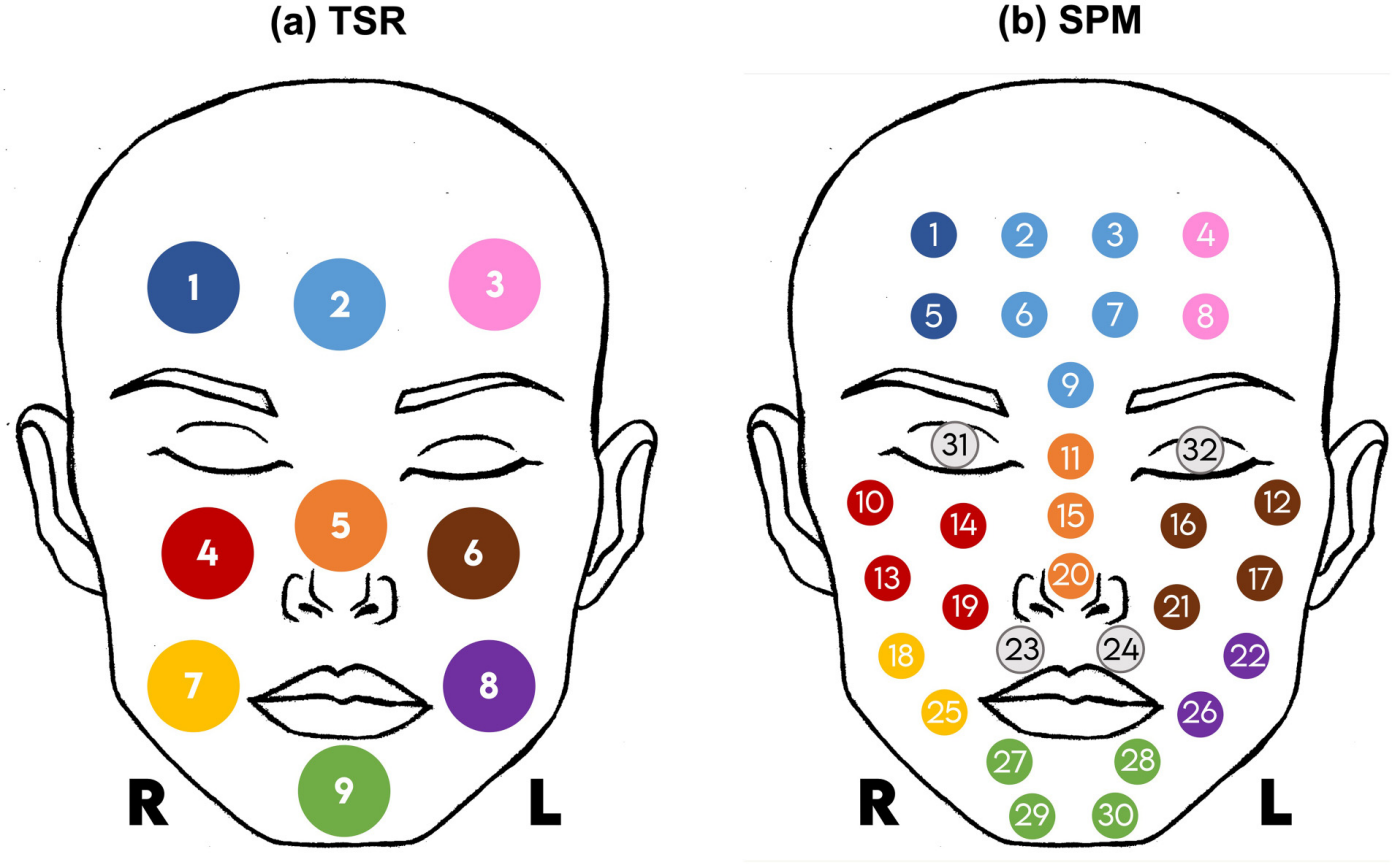
Location of the facial measurements for TSR and SPM instruments, with color-coding used to relate the area of measurements when using each instrument. “R” and “L” refer to the right and left sides, respectively. (a) Location of the nine facial measurements using the TSR. (b) Location of the 32 facial measurements using the SPM. Measurements 31 and 32 were taken over the eyelids, with the eyes closed.

A PR-650 telespectroradiometer (Spectra Scan Colorimeter, PhotoResearch Inc., USA) was used to measure the spectral radiance from nine facial locations as indicated in Figure 1a. The color coding was used to relate these areas to the areas of acquisition when using a contact spectrophotometer (SPM) presented in Figure 1b.

The participants were 75 cm from the instrument, resulting in a field of view of approximately 1°, corresponding to an area of about 1.4 cm^2^. The spectral range considered was from 380 to 780 nm, in 4 nm steps. The light source was a 150 W metal halide lamp (Osram, Powerstar, HQI-TS 150W/NDL, Germany), placed in front of the participant's face at about 75 cm, producing a luminance of about 3250 cd/m^2^ measured with a lux meter (Illuminance meter T-10, Konica Minolta Co., Ltd., Japan) at the face position. To avoid natural facial movement during measurements the participants were leaning against a head support.

Radiance was measured at each location of the facial skin, as presented in Figure 1a, and a barium sulfate (BaSO_4_) sample was placed at the measurement location instead of the facial skin. Spectral reflectance was estimated from Eq. 1 ^29,97^ considering that the measured spectral radiance ( β(λ) ) is proportional to the product between the spectral power distribution of the illuminant ( S(λ) ) and the spectral reflectance ( R(λ) ):

(1) β(λ)∝R(λ)S(λ)

Assuming that the radiance spectrum of the skin ( $\beta (\lambda {)}_{\mathrm{skin}}$ ) and the radiance spectrum and the illuminant [BaSO_4_ ( $\beta (\lambda {)}_{{\mathrm{BaSO}}_{4}}$ )] correspond to the measured spectra, the reflectance spectrum was obtained using Eq. 2.

(2) $$R(\lambda )\propto \frac{\beta (\lambda )}{S(\lambda )}\propto \frac{\beta {\left(\lambda \right)}_{\mathrm{skin}}}{\beta {\left(\lambda \right)}_{{\mathrm{BaSO}}_{4}}}$$

### Spectrophotometric Measurements

A spectrophotometer (spectrophotometer CM-2600d, Konica Minolta Co. Ltd., Japan) was used to measure the skin reflectance using a small aperture mask (SAV) with an area of around 7 mm^2^, smaller than the area measured using the TSR. To prevent direct contact with the skin, the instrument was wrapped in a transparent plastic bag, for each new participant. Thirty-two measurements were taken at locations presented in Figure 1b.

The same color code was used in Figure 1a and b to identify the location of the measurements of the SPM and TSR, for further analysis. The SPM considered smaller measurement areas than the TSR, so the average of a set of points was considered and represented with the same color for reference.

### Hyperspectral Imaging Measurements

The acquisition of the hyperspectral data was carried out using a custom-built hyperspectral imaging system, described in detail elsewhere.^98–102^ It was composed of a Peltier-cooled low-noise digital monochromatic camera with a spatial resolution of 1344 (horizontal) × 1024 (vertical) pixels and a 12-bit output precision (Hamamatsu, Model C4742-95-12ER, Hamamatsu Photonics K.K., Japan). The charge-coupled device (CCD) had a physical dimension of 8.66 × 6.66 mm, with a pixel size of 6.45 × 6.45 μm. A focusing lens was attached in front of the camera with the aperture set to *f*/11, maximum zoom, and a focal distance between 12.5 and 75 mm, producing about 1′ of acquisition field per pixel. A fast tunable liquid-crystal filter (Varispec, Model VS-VIS2-10-HC-35-SQ, Cambridge Research and Instrumentation, Inc., USA) was placed in front of the lens, together with an infrared blocking filter to avoid infrared light contamination when the system was tuned for low wavelengths. Data were acquired from 400 to 720 nm in 10 nm steps, with a half-height bandwidth of 7 nm, 10 nm, and 16 nm at 400 nm, 550 nm, and 720 nm, respectively.

The accuracy of the HIS in recovering spectral reflectance functions was found to have an average spectral difference of 2%,^102^ with an average colorimetric error of 1.3 when estimated assuming the CIEDE2000 ( $\Delta E_{00}*$ ) color difference formula,^103^ and of 2.2 if estimated assuming the CIELAB (ΔE*ab) color difference formula,^97^ in close agreement with the chromatic discrimination threshold for complex images.^104^

**Figure 2.**
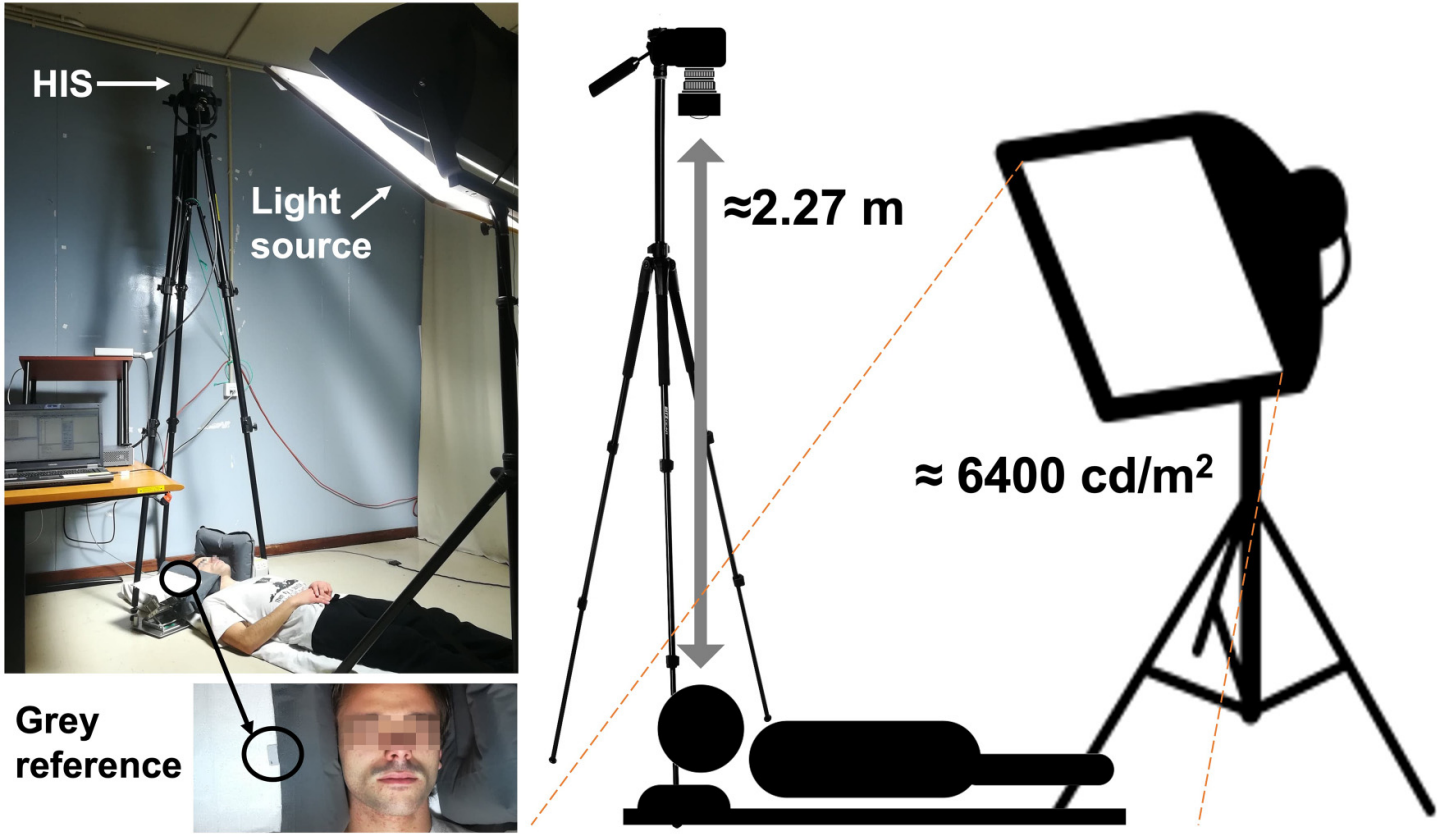
Setup for the acquisition of the hyperspectral images, highlighting the positions of the participant, the camera, and the light source with the UV filter in front. The participant was lying on the floor and the participant's head was supported by pillows. The image's inset highlights the gray reference that was placed at the face level, visible during the time of acquisition.

Figure 2 presents the setup used for the acquisition of the facial spectral data, using the HIS. The system was mounted vertically and towards the floor. Participants were positioned lying down on the floor, under the HIS, facing the system, at approximately 2.27 m. The participant's face was illuminated by a 1000 W metal halide lamp (Osram, Powerstar, HQI-T, 1000W/D, Germany) using an ultraviolet (UV) blocker filter in front of the lamp for protection from the UV radiation produced by the lamp. A luminance of approximately 6400 cd/m^2^ was available at the face level, measured using a lux meter (Illuminance meter T-10, Konica Minolta Co., Ltd., Japan). At the time of acquisition, participants had their heads blocked on the sides with supporting pillows to prevent head movements and were instructed to have their eyes and lips closed, and a neutral face expression, while avoiding movements. The neutral facial expression was adopted for practical reasons and to be more comfortable for the participant during the acquisition process. Also, as the focus of the images obtained was on the color of the skin, white areas such as the teeth and the eye globes were avoided. A Munsell N7 gray reference was placed next to the face for calibration purposes, as can be seen in Figure 2.

Before each image acquisition, to guarantee optimal signal output, exposure times were estimated for each wavelength individually ensuring a digital output of 85% of the maximum signal available.

Reflectance data ( R(λ) ) were computed for each image pixel using Eqs. 3 and 4. Radiance data ( β(λ) ) for each pixel were computed using Eq. 3, considering $\beta_{N7}(\lambda )$ as the radiance from the gray reference measured using the TSR at the time of acquisition, $\beta_{\mathrm{reference}}(\lambda )$ the average signal from a selected area of the reference presented in the scene and acquired with the HIS and $\beta_{\mathrm{image}}(\lambda )$ the signal of each pixel as acquired with the HIS. Data computations were performed after correcting the data acquired for dark noise and stray light.

Reflectance data ( R(λ) ) was then computed from the radiance data ( β(λ) ) using Eq. 4, assuming that the illuminant data ( S(λ) ) were acquired with the HIS and extracted from an area of the gray reference after compensating for illuminant spatial nonuniformities by using a white uniform reference. The spectral acquisition of a flat white uniform reference returned the radiance of the illuminant spatial variation that was used to compensate for the illuminant nonuniformities at the facial level.

(3) $$\beta (\lambda )=\beta_{\mathrm{image}}(\lambda )\times \frac{\beta_{N7}(\lambda )}{\beta_{\mathrm{reference}}(\lambda )}\;$$

(4) R(λ)=β(λ)/S(λ)

Data were processed using Matlab with the image processing and imaging acquisition toolboxes (The MathWorks, Inc., USA).

During the acquisition process, great care was taken to minimize any movement of the participant's head and face. Nevertheless, in some cases, participants made small eyelid or lip movements, which resulted in local image artifacts (as presented in Figure S2, Supplemental Material). These images were preserved in the database but tagged with areas with movement.

Selecting individual wavelengths for visualization highlights specific facial features that are not visible when resorting to traditional red-green-blue (RGB) colored images. To observe more details of facial features at each wavelength (see Figure S3, Supplemental Material).

### Analysis of Spectral Reflectance and Chromatic Content

To analyze the spectral reflectance across the different techniques, TSR, SPM, and HIS, a subsample of 16 out of the total of 29 faces from the database was considered. To obtain comparable areas of analysis, multiple SPM measures were combined as color-coded in Figure 1. Reflectance data estimated from the skin of the area identified as 1 (as presented in Figure 1a) were compared with the reflectance data estimated by averaging reflectance data from the skin across areas 1 and 5 (as presented in Figure 1b, proceeding equivalently for the other color-coded areas. The SPM reflectance data from the skin were, therefore, averaged from the 32 available data points (as presented in Figure 1b) into the nine areas (as presented in Figure 1a), considering the surrounding data points and following the color code. HIS provided a higher number of reflectance data from the skin, which forced a different methodology to estimate the average reflectance to obtain comparable data to compare HIS with SPM and TSR. Reflectance data from the facial skin obtained using the HIS were averaged across a collection of reflectance spectra made to match a circular area with a diameter of about 100 pixels, selected to correspond to the nine areas as presented in Figure 1a, averaging a total of about 7500 reflectance spectra into only one reflectance spectra representative of the area under analysis. Special care was taken to select areas that only enclosed facial skin, without other facial features (e.g., hair, eyes, or lips). Data from the HIS were also used to compare the left and right sides of the face, for all faces considered, and Groups 1 and 2 separately, to investigate if the human faces have uniform reflectance spectra across the left and the right facial side.

To ascertain the spectral variations across the reflectance spectra of the skin obtained by using the HIS, TSR, and SPM methodologies two metrics were used, the root mean square error (RMSE), as defined in Eq. 5, and the chromatic difference, ΔE*_(L*, a*,b*)_ (corresponding to the CIE (ΔE*_ab_)^97^ and ΔE*_a*,b*_, as defined in Eqs. 6 and 7, respectively. The chromatic differences were estimated in the CIELAB color space assuming the CIE D65 illuminant and the CIE 2006 10° cone-fundamental-based colorimetric observer.^97^

To ascertain the spectral variations across the reflectance spectra of the skin obtained by using the HIS, TSR, and SPM methodologies two metrics were used, the RMSE as defined in Eq. 5, and the chromatic difference, ΔE*_(L*, a*,b*)_ and ΔE*_a*,b*_, as defined in Eqs. 6 and 7, respectively. The chromatic differences were estimated in the CIELAB color space assuming the CIE D65 illuminant and the CIE 2006 10° cone-fundamental-based colorimetric observer.^97^

RMSE was computed considering the sum of the difference between the reflectance data and the minimum reflectance data of the sample under analysis (ΔR(λ)) over the wavelength range ( λ ), for the number of reflectance spectra considered ( N ), across all pixels of the sample, expressed by Eq. 5:

(5) $$RMSE=\sqrt{\frac{\sum_{\lambda \;}\;{\left(\Delta R(\lambda )\right)}^{2}}{N}}$$

The chromatic differences were computed by estimation of the chromatic differences for ΔE*_(L*, a*,b*)_ that included the L*, a*, b* chromaticity coordinates of comparing colors (as defined in Eq. 6), and the chromatic differences for (ΔE*_a,b_) that included only a* and b* chromaticity coordinates of comparing colors (as defined in Eq. 7), assuming the CIELAB color space.^97^

(6) $$\Delta E_{\left(L*,a*,b*\right)}*=\sqrt{{\left(\Delta L*\right)}^{2}+{\left(\Delta a*\right)}^{2}+{\left(\Delta b*\right)}^{2}}$$

(7) $$\Delta E_{\left(a*,b*\right)}*=\sqrt{{\left(\Delta a*\right)}^{2}+{\left(\Delta b*\right)}^{2}}$$

The color volume created by all the chromaticity coordinates estimated from the reflectance data acquired with the HIS system was estimated by counting the number of discernible colors, when representing the number of just noticeably different (JND) colors available in the color volume, by segmenting the color volume into unitary cubes and by counting the nonempty cubes, assuming that all the colors that were inside the same cube could not be discernible.

## Results and Discussion

### Spectral and Colorimetric Analysis of the Hyperspectral Images

The spectral reflectance and the CIELAB chromaticity coordinates estimated for each participant of the HIS hyperspectral images database were divided into two groups (Groups 1 and 2) and averaged across participants of the same group and for each facial position across positions 1 to 9, to study the influence of the skin tone and the area of the skin under analysis.

**Figure 3.**
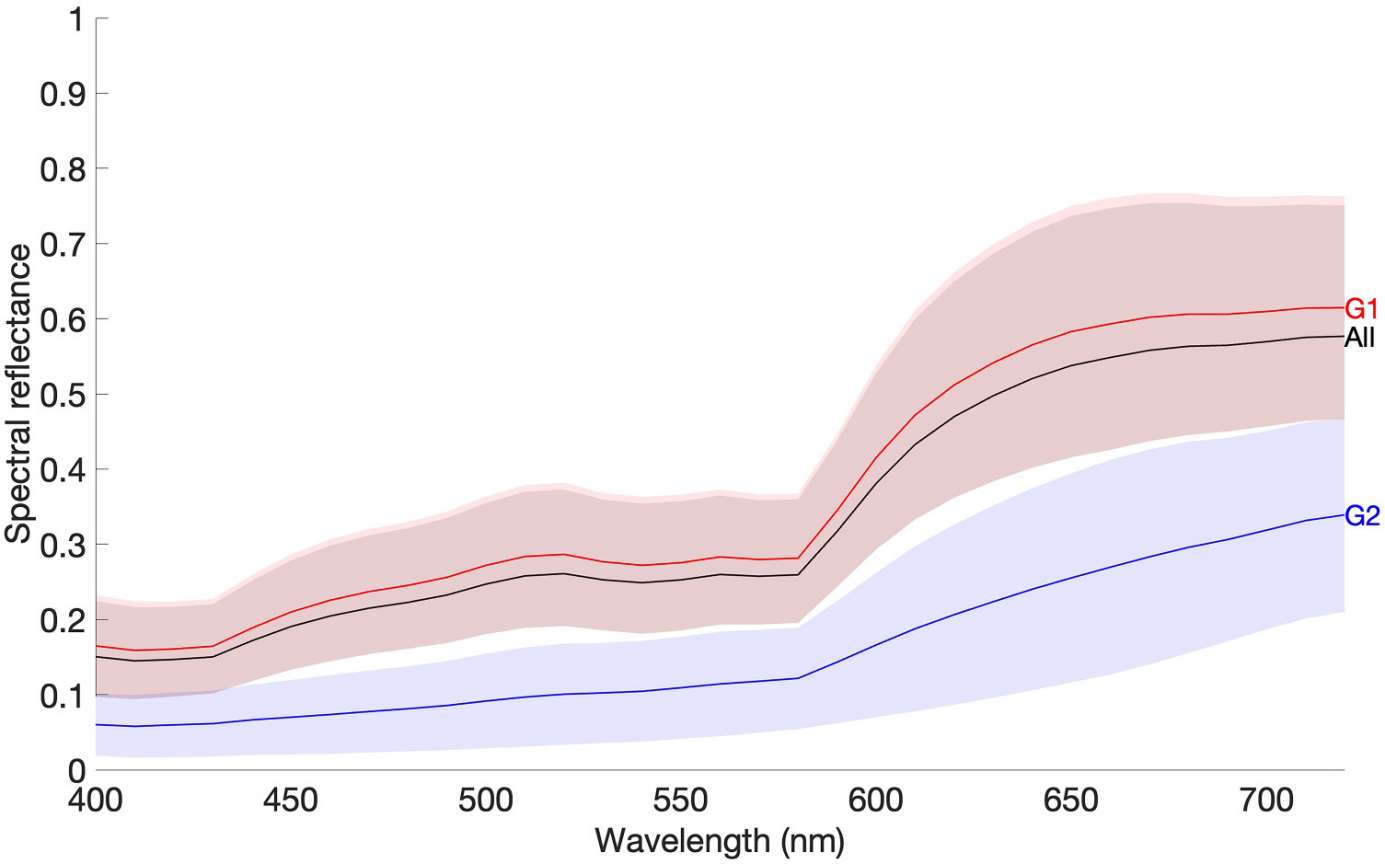
Average spectral reflectance (solid line) and standard deviation (shaded area) across all nine facial positions from all 29 faces from the HIS database (“all”, black line and shaded area), and across faces from Group 1 (G1, red line and shaded area) and Group 2 (G2, blue line and shaded area).

Figure 3 presents the average (solid lines) and standard deviation (shaded areas) of the reflectance spectra across all 29 faces (all represented by the black line and the gray shaded area), and faces classified as Group 1 (G1 represented as the red line and the reddish shaded area) and Group 2 (G2 represented as the blue line and the bluish shaded area), for all the pixels from all nine facial positions. The red-shaded area from the faces of Group 1 mostly overlapped with the gray area representative of all 29 faces, as expected from the lower number of participants in Group 2, resulting in an unexpressive contribution of this group to the overall average.

Comparing facial positions, variability between them was revealed with positions 2 (P2), 4 (P4), 6 (P6), and nine (P9) (corresponding to forehead center, and right and left cheeks and chin, respectively, as presented in Figure 1a) having higher reflectance values than the average for Groups 1 and 2, with Group 2 showing lower reflectance values overall when comparing to Group 1. (To visually observe these differences between facial positions at each skin group, see Figure S4, Supplemental Material).

**Figure 4.**
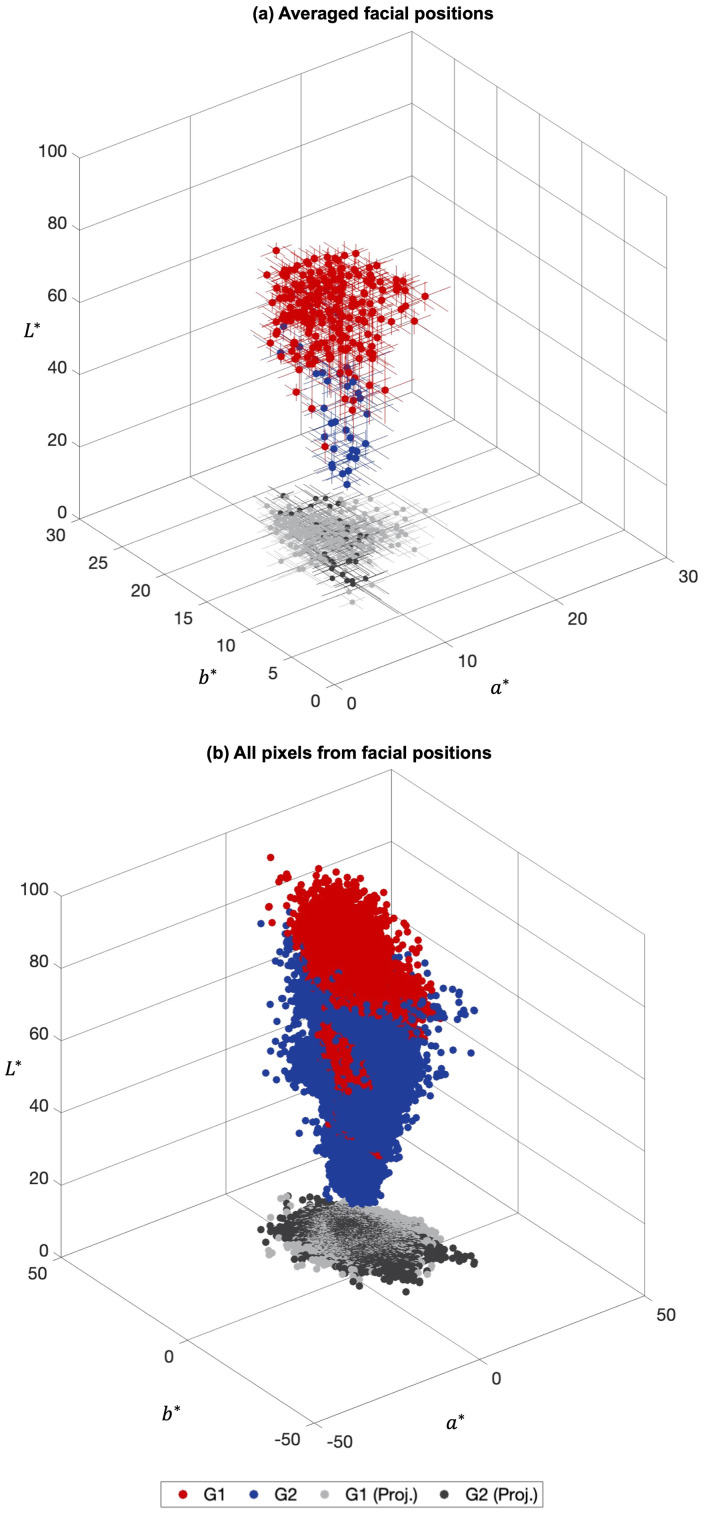
CIELAB chromaticity coordinates estimated from the spectral reflectance of the 29 faces database for the nine facial positions for Group 1 (G1) and Group 2 (G2) faces, represented as red and blue, respectively, with their CIE (a*, b*) projections represented as light gray and dark gray, respectively. (a) Represents data averaged across all pixels for each facial position and face resulting in one chromaticity coordinate per facial position, and (b) represents all the CIELAB chromaticity coordinates estimated from the same nine areas considered in (a) when considering the entire information available from the hyperspectral images. Please note that plots (a) and (b) were drawn with different a* and b* scales.

When converting reflectance to CIELAB color coordinates considering a unique CIELAB coordinate as representative of the data found in the corresponding areas of HIS images, the local chromatic variation available will reduce significantly. Figure 4 presents CIELAB chromaticity coordinates across facial positions for Group 1 (G1, red dots) and Group 2 (G2, blue dots), with the corresponding projections in CIE (a*, b*) (as light gray for G1 and dark gray for G2). Figure 4a presents the average (dots) and standard deviation (lines) across all pixels of each one of the nine facial positions identified in Figure 1a, for the 29 participants, resulting in nine chromaticity coordinates per face. Each coordinate in Figure 4b represents the CIELAB chromaticity coordinate estimated from the same nine areas of the faces but considering not the average across pixels per area, as presented in Figure 4a, but considering all pixels that represented each one of the nine facial positions for all the 29 faces of the database (each facial position was represented by 100 pixels in diameter, resulting in about 7500 pixels per area under analysis). Differences were found between the volume occupied by the chromaticity coordinates in Figure 4a and 4b, highlight the amount of information lost when considering only single-point measurements or estimations of the average color.

The average CIELAB chromaticity coordinates and standard deviation (estimated across all chromaticity coordinates presented in Figure 4b considering each group separately, the nine facial positions, and the 29 faces of the database) were 61.8 ± 8.8, 12.9 ± 2.7, 17.5 ± 3.1 for Group 1, and 39.4 ± 10.8, 12.6 ± 2.7, 16.4 ± 4.7 for Group 2, for L*, a*, b*, respectively. The average difference between Group 1 and Group 2 was ΔE*_(L*, a*,b*)_ ≈ 22 units, and (ΔE*_a,b_) ≈ 1.7 units when $L^{*}$ is not considered.

When comparing the plots in Figure 4a and 4b, the increase in the number of the blue and red chromaticity coordinates from Figure 4a and 4b present the increase in the amount of available spectral information if the HIS system was used instead of punctual spectral measuring systems (as the SPM and the TSR devices).

Considering CIELAB chromaticity coordinates segmented for each one of the nine facial positions individually, presented in Figure S5, (Supplemental Material), differences in the CIELAB color volumes across positions are noticeable, with position 2 (central forehead) and position 5 (nose) having higher color volume (1.5 and 1.4 times more (for positions 2 and 5, respectively) than the highest remaining color volume estimated by the number of discernible colors across all observers) than the remaining positions for all participants, for Group 1 (1.2 and 1.5 times for positions 2 and 5, respectively), and for Group 2 (1.7 and 1.1 times for positions 2 and 5, respectively).

**Figure 5.**
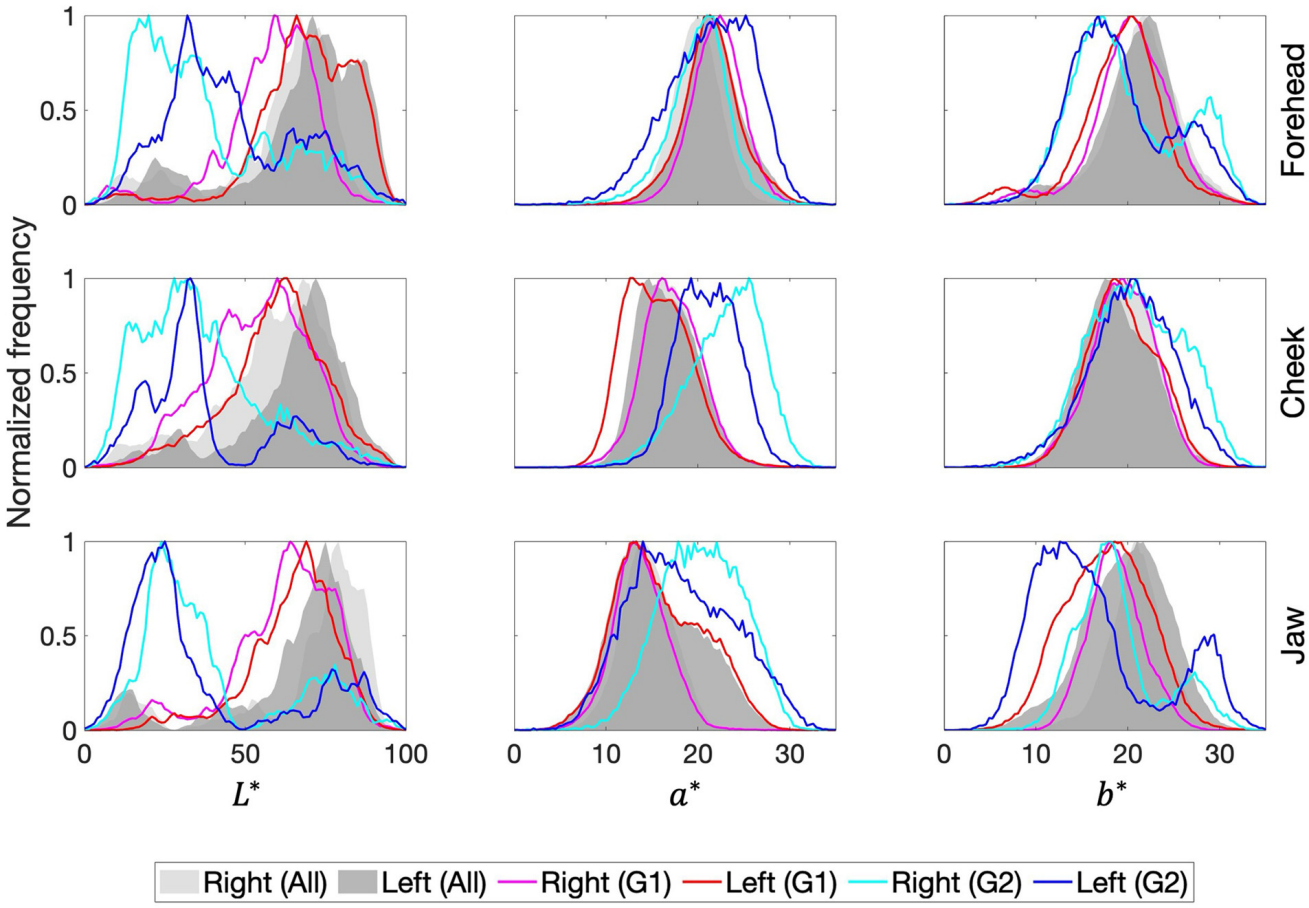
Normalized frequency distribution of CIE L* (left column), a* (center column), and b* (right column) chromaticity coordinates, for the forehead (top panel, comparing positions 1 and 3), the cheekbones (center panel, comparing positions 4 and 6), and the jawline (bottom panel, comparing positions 7 and 8). Shaded areas represent data across all observers (dark gray for the left facial side and light gray for the right facial side), while lines represent Group 1 (magenta line for the right facial side and red line for the left facial side) and Group 2 (cyan line for the right facial side and dark blue line for the left facial side).

The variations in the color volume found for the nine facial positions (presented in Figure S5, Supplemental Material) are not the only variations that should be highlighted when considering the chromatic diversity found when analyzing hyperspectral images of faces. Differences were found for the left and the right side of the faces and for Group 1 and Group 2. Figure 5 presents the normalized frequency distribution of the CIELAB chromaticity coordinates (L* at the left column, a* at the central column, and b* at the right column) for three facial zones for the right and left side as presented in Figure 1a, forehead (position 1 compared to position 3), cheek (position 4 compared to position 6), and jawline (position 7 compared to position 8). Shaded areas represent chromaticity coordinates across all observers (dark gray for the left facial side and light gray for the right facial side), while lines represent Group 1 (magenta line for the right facial side and red line for the left facial side) and Group 2 (cyan line for the right facial side and dark blue line for the left facial side). It was found the distribution of the normalized frequencies was not the same across facial positions when comparing the right and the left facial sides and the different facial areas, in particular when considering Group 1 and Group 2 independently.

Table II presents the position of the maximum of each normalized frequency distribution of CIELAB chromaticity coordinates presented in Figure 5. When considering the average chromaticity coordinates across positions 1, 4, and 7, and across positions 3, 6, and 8, the ΔE*_(L*, a*,b*)_ between these two averaged chromaticity coordinates was found to be 5.6 ± 1.3 for Group 1 and 9.1 ± 3.2 for Group 2, while the (ΔE*_a,b_) was found to be 1.7 ± 1.6 for Group 1 and 5.7 ± 1.3 for Group 2. These results seem to indicate a high influence of the L* chromaticity coordinate in the chromatic difference between sides, and differences are higher for Group 2 than for Group 1.

**Table II.** CIELAB chromaticity coordinates corresponding to the position of the maximum of each normalized frequency distribution for L*, a*, and b* chromaticity coordinates, for each facial position (correlated with the facial zone), facial side, and for Group 1 and Group 2. Data were rounded to the closest integer.

Face			Group 1			Group 2		
Zone	Side	Position	L*	a*	b*	L*	a*	b*
Forehead	Right	1	59	22	20	20	21	17
	Left	3	66	21	20	32	25	17
Cheek	Right	4	60	16	19	28	26	21
	Left	6	63	13	19	33	19	21
Jawline	Right	7	64	13	18	24	18	18
	Left	8	69	13	19	25	14	13

The average color difference ΔE*_(L*, a*,b*)_ between the left and right positions 1 and 3, positions 4 and 6, and positions 7 and 8, was found to be 5.5 ± 1.2 for Group 1 and 9.2 ± 2.5 for Group 2, while the (ΔE*_a,b_) was found to be 1.7 ± 0.9 for Group 1 and 5.8 ± 1.3 for Group 2. These results seem to indicate that the color differences found between facial positions of the same facial side (considering positions 1, 4, and 7, and positions 3, 6, and 8) and on the left and right side of the face (considering positions 1 and 3, positions 4 and 6, and positions 7 and 8) have an equivalent magnitude of color differences.

**Figure 6.**
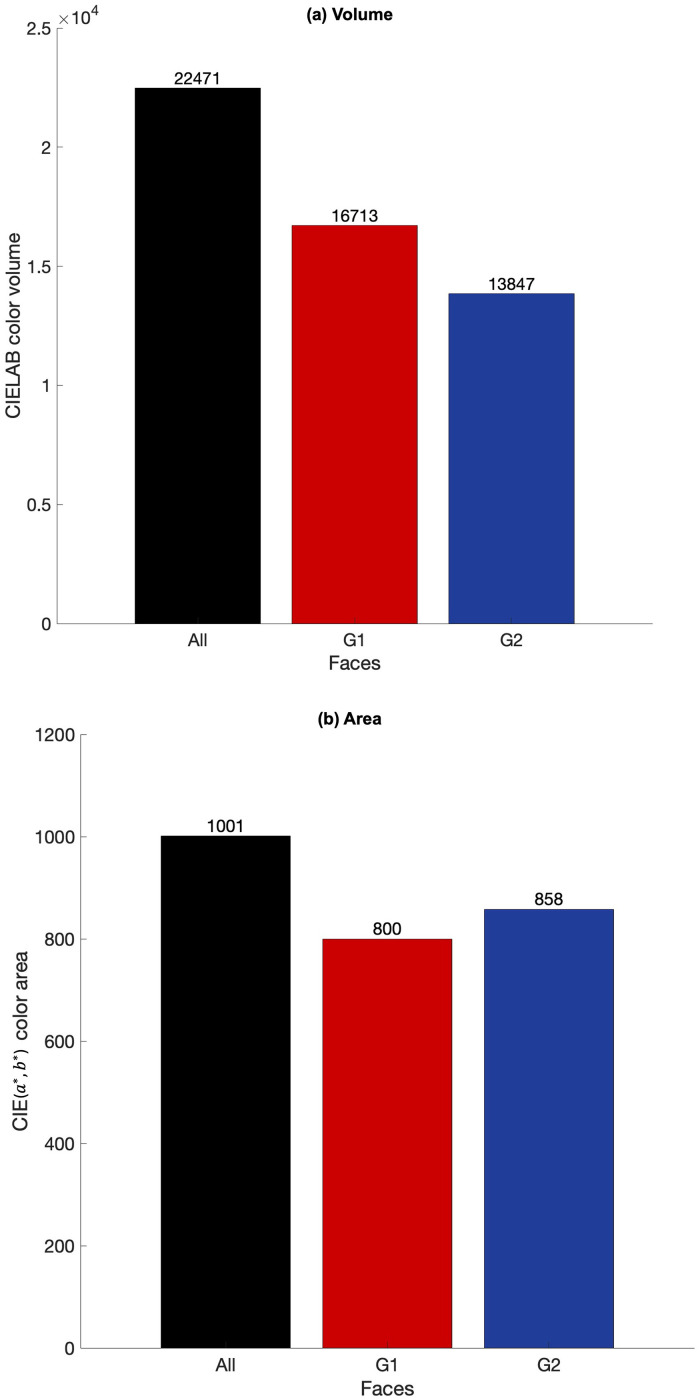
(a) CIELAB color volume and (b) CIE (a*, b*) area estimated by counting the number of discernible colors, for all 29 faces including only the nine facial positions under analysis (“all”, black bars), all faces of Group 1 (G1, red) and all faces of Group 2 (G2, blue).

Figure 6 presents the computations of the CIELAB color volume (Figure 6a) and CIE (a*, b*) area (Figure 6b) for all the chromaticity coordinates of all 29 faces (“All”, black bars) including all the nine facial positions, for Group 1 faces (G1, red bars) and for Group 2 faces (G2, blue bars), assuming the number of discernible colors as an estimation of the color volume. The number of discernible colors computed referred to the CIELAB color volume presented in Figure 4b. It was found that the color volume produced by the faces of Group 1 does not overlap with the color volume produced by Group 2, as estimating the total color volume of the combination of both chromaticity coordinates of the faces of both groups yields a higher color volume than each group alone, and as demonstrated by the differences between the average CIELAB chromaticity coordinates presented earlier, with higher luminance variations (ΔE*_(L*, a*,b*)_) than chromaticity variations (ΔE*_(a*,b*)_). When considering the color volume, and despite the differences in the relative number of participants in Group 1 and Group 2, Group 1 has a higher color volume, but Group 2 has only a marginal higher color area when estimating the number of discernible colors and ignoring the L* dimension.

**Figure 7.**
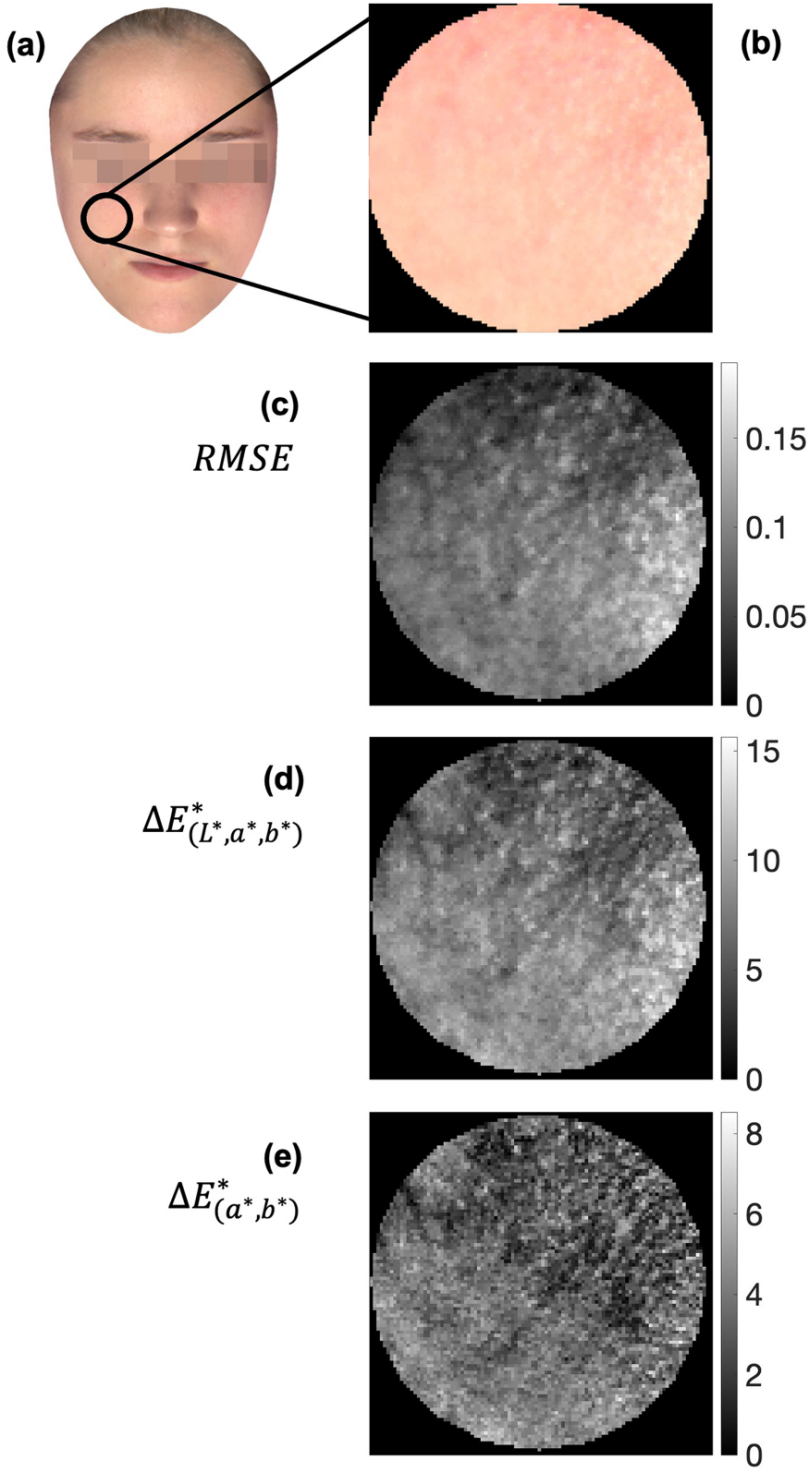
(a) Spectral and color variance over an area of facial skin from a participant from Group 1 with a diameter of about (b) 100 pixels color rendering of the spectral data retrieved from the hyperspectral image of the patient's face). (c) The RMSE variations from the minimum spectral reflectance against the spectral reflectance retrieved from each pixel. (d) The color variations in ΔE*_(__L__*, a*,b*)_ units from the minimum CIELAB color coordinates against the CIELAB color coordinates estimated from each pixel, with (e) representing the same data considering only a* and b* chromaticity coordinates, ΔE*_(__a__*,b*)_. In panels (c), (d), and (e), black colors represent no difference, while white colors represent the maximum difference.

The spectral and chromatic variations were also analyzed considering local areas. Figure 7 presents the spectral and color variance found in an area of the skin of a face from Group 1 (Figure 7a) when the data of the HIS were considered. Figure 7b presents color rendering of the data from the hyperspectral image of the area analyzed, representing about 100 pixels in diameter (with a diameter of about 16.9 mm or 2.24 cm^2^ encompassing about 7500 pixels), equivalent to approximately 1° of visual angle if the face was viewed at 1 m). This area of analysis was selected to be similar to the field of view of the TSR, with an area of about 1.4 cm^2^ for a viewing distance of 75 cm (Figure 1a), but bigger than the measurement area of the SPM of about 7 mm^2^. Figure 7c presents the RMSE estimated between the minimum reflectance spectrum and the reflectance spectrum found at each image pixel, as an estimation of the spectral variation found when considering a facial area of about 1° of the visual field. It was found that variations ranged from 0.03 (min) to 0.3 (max) with an average of 0.1 ± 0.1. While the RMSE provides a quantitative variation across the area analyzed, it does not provide a perceptual variation associated with the spectral difference. Figure 7d presents the ΔE*_(L*, a*,b*)_ estimated between the minimum CIELAB color coordinate and the CIELAB color coordinates estimated for all other image pixels in the area of analysis. It was found that the CIELAB variations ranged from 4.0 (min) to 27.7 (max) with an average of 11.1 ± 5.4. If the effect of the $L^{*}$ dimension is ignored, it was found that the ΔE*_(a*,b*)_ variations range from 1.4 (min) to 15.5 (max) with an average of 4.5 ± 2.8, as presented in Figure 7e.

### Spectral Reflectance Acquired Using Different Methodologies

**Figure 8.**
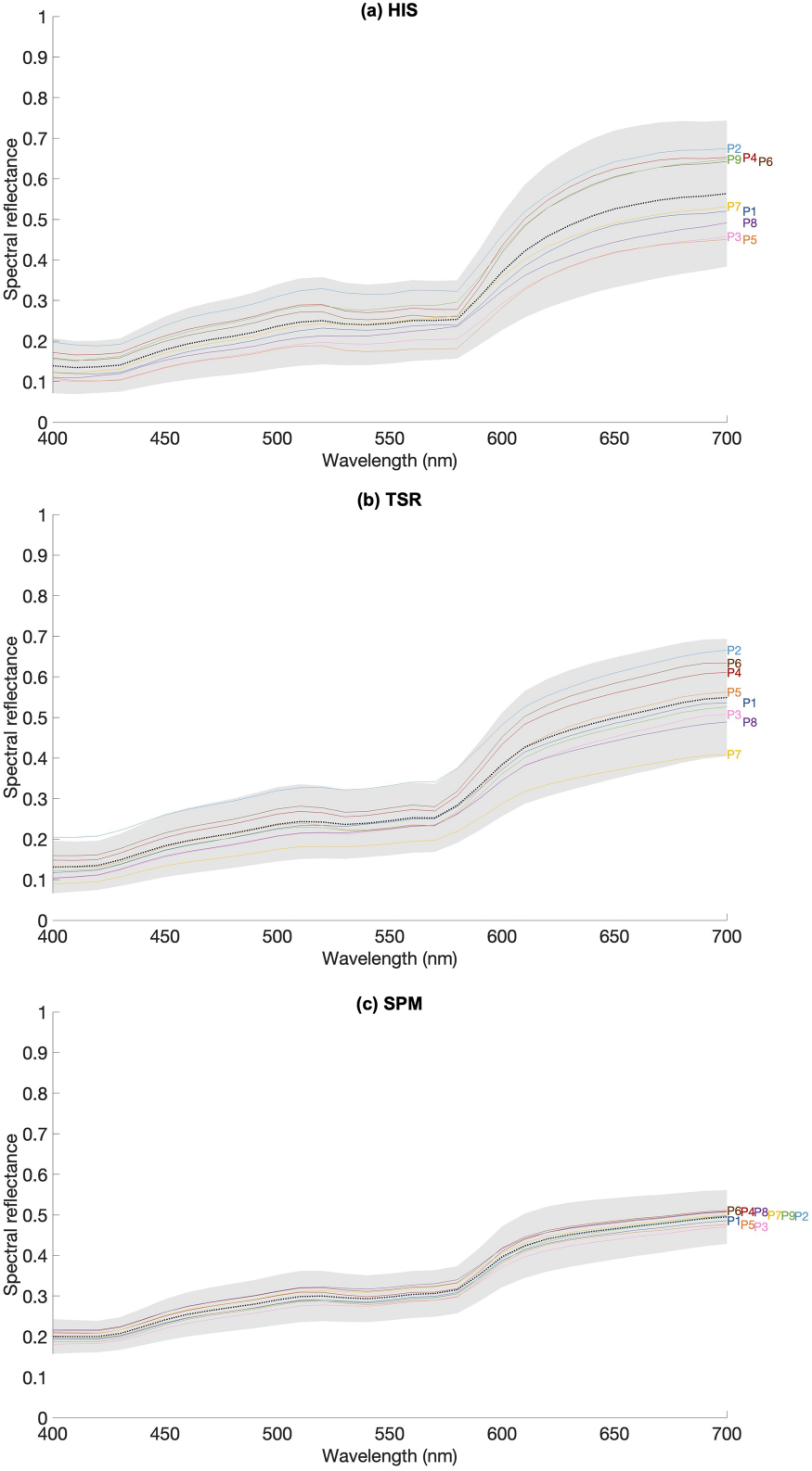
Average (dotted line) and standard deviation (shaded area) for spectral reflectance obtained with the (a) HIS, (b) TSR, and (c) SPM across 16 faces for each facial position (P1 as position 1 to P9 as position 9) represented as colored lines (using the same color code as Figure 1).

Figure 8 presents the average (dotted black line) and standard deviation (shaded area) of spectral reflectance as a function of wavelength measured with HIS (Figure 8a), TSR (Figure 8b), and SPM (Figure 8c). Numbers 1 to 9 indicate the position measured on the face as shown in Figure 1, represented by solid lines with the corresponding color-coding. Data from 16 faces (a subset from the 29 faces) are presented from 400 to 700 nm in 10 nm steps, after data interpolation, in the case of the TSR data, for comparison.

Table III presents the average and standard deviation of the RMSE, and the ΔE*_(L*, a*,b*)_ values estimated between the reflectance data obtained using the HIS and with the SPM (HIS versus SPM) and with the TSR (HIS versus TSR) across 16 faces of the database, considering each facial position from position 1 to position 9 as presented in Figure 1a. The single reflectance spectra extracted from the HIS for each facial area were estimated by averaging the spectral reflectance across all pixels of the area analyzed. The data were interpolated to adjust the spectral reflectance from 400 to 700 nm in 10 nm steps. It was found that the RMSE data were similar across conditions when having the HIS as the comparison. Nevertheless, similarities in the spectra shape did not have the same correspondence to the differences in the perceived color, as the CIES ΔE*_ab_ data were lower when comparing the HIS with the TSR than when comparing the HIS with the SPM (on average 11.61 ± 1.81 and 13.96 ± 1.19, respectively). When comparing the average spectral reflectance presented in Figure 8, the average spectral reflectance obtained when using the SPM had a higher intensity when compared to HIS and the TSR. Such results seem to indicate that while the shape profile of the reflectance spectra seems equivalent across acquisition devices, the computed perceived color obtained using the HIS has higher similarity with the data acquired using the SPM than using the TSR.

**Table III.** Average and standard deviation of RMSE and ΔE*_(__L__*, a*, b*)_ estimated between the spectral reflectance data acquired using the HIS and the TSR, and the SPM for each of the nine facial positions across 16 faces of the database.

		RMSE		ΔE*_(__L__*, a*,b*)_		
		HIS versus SPM	HIS versus TSR	HIS versus SPM		HIS versus TSR
Position	1	0.11 ± 0.06	0.11 ± 0.08	13.93 ± 7.83	11.91 ± 9.02	
	2	0.15 ± 0.05	0.15 ± 0.07	12.47 ± 3.77	11.92 ± 6.00	
	3	0.12 ± 0.07	0.11 ± 0.07	14.81 ± 9.17	12.40 ± 8.99	
	4	0.12 ± 0.05	0.11 ± 0.07	12.24 ± 4.52	9.76 ± 5.30	
	5	0.11 ± 0.06	0.10 ± 0.05	14.07 ± 6.57	10.16 ± 5.23	
	6	0.12 ± 0.05	0.10 ± 0.07	13.51 ± 5.00	8.98 ± 6.04	
	7	0.12 ± 0.08	0.13 ± 0.08	15.05 ± 8.69	13.09 ± 7.22	
	8	0.13 ± 0.08	0.10 ± 0.08	15.92 ± 9.14	11.43 ± 7.74	
	9	0.15 ± 0.07	0.17 ± 0.10	13.65 ± 6.56	14.87 ± 10.95	
Average		0.13 ± 0.01	0.12 ± 0.02	13.96 ± 1.19	11.61 ± 1.81	

The purpose of this work was to provide a cured and validated database of hyperspectral images of human faces, describe the acquisition process, and an analysis of the spectral and colorimetric features.

 Overall, the average facial skin color tends towards red-yellow tones independently of the skin tone group considered. The reflectance spectrum and the CIELAB chromaticity coordinates agree with previous reports.^19–21,29,33,44,46,72,93–96^ The CIELAB color space was selected for this analysis as it offers a practical and precise way to identify and communicate skin color information for clinical and scientific purposes.^42,105,106^

The spectral reflectance obtained for Group 2 was lower than that of Group 1 (Figure 3 and Figure S4, Supplemental Material) resulting in lower CIE L* values (Figure 4) as expected from data published elsewhere.^20,23,27–29,33^ Such findings were extended to any facial position considered (Figure 5 and Figure S5, Supplemental Material) and were related to the concentration of melanin in the skin (higher for Group 2 than for Group 1). The increase in the concentration of melanin in the skin reduced the instrument's capability to measure the hemoglobin spectral reflectance. Such a result is of importance as it was found that the role of L* in the estimation of the chromatic difference is higher than the chromatic differences alone. These findings agree with the evidence that there is an inverse correlation between the melanin index and the L* chromaticity coordinate.^107^

Furthermore, the increased color gamut, when considering image pixels individually as presented in Figure 4b, against the much-reduced color gamut as presented in Figure 4a, supports the advantage of using hyperspectral imaging to obtain spectral information of human facial skin. Figure 5 also highlights the fact that a single descriptor of the facial side or position was not enough for a full description of the distribution of the chromaticity coordinates across a human face.

Such results may be of importance when attempting to simulate the spectral profile of the human skin, as the variability of the spectral information seems to be high across the entire face and even across a small area.^108,109^ As seen in Figure 7, local chromatic variations can achieve chromatic differences of around 11 units or around five units if L* is not considered, which are important chromatic variations^110^ that may provide relevant information in opposition to when perception is based on simulations of facial color retrieved from TSR measures.^14^ If a single spectrum is selected to represent the area analyzed, chromatic errors of visual importance and perceived even in images of complex scenarios will be obtained, as described elsewhere that the chromatic threshold for complex images is about 2.2.^104^ These errors still hold if different facial positions are considered (as presented in Figure 5 and Figure S5, Supplemental Material) where differences are highlighted by the differences across color volumes estimated for each facial position analyzed. These local variations should not be disregarded as local color perception associated with local facial features might intensify specific features and/or provide different facial perceptions.^111^

These results still hold if other methodologies are used to estimate the difference between the HIS data of a facial area position and a single spectrum representative of such an area. The goodness of fit (GOF) and the color difference estimated in the SCIELAB color space (ΔE*_SCIELAB_) were considered.^112–114^ The GOF fit was estimated by using the “GoodnessOfFit” function with the NRMSE option selected, from the system identification toolbox in Matlab v.2024a. The SCIELAB color space was used to estimate the color differences assuming the colored pixel in the context of the image and the spatial and chromatic sensitivities of the human eye, and not using the individual pixel as was done across this paper. SCIELAB compares images and, when required, an image with the same size as the facial area under analysis was built, assuming a replication of a single reflectance spectrum per the number of pixels. Colored images were rendered from the spectral data by assuming the CIE 1931 2° standard observer and CIE D65 illuminant, converting the spectral reflectance into the *x*, *y*, and *z* tristimulus values. The single reflectance spectrum acquired using the TSR, the SPM, or, as a reference, the minimum spectral reflectance of the data acquired with the HIS (minHIS, as presented in Figure 7) was compared to the reflectance data of the same facial area as acquired with the HIS. The GOF difference between the TSR and the HIS was 1.3 ± 0.4, between the SPM and the HIS was 1.9 ± 1.8, and between the minHIS and the HIS was 1.1 ± 0.1, with values representing the average and the standard error estimated across the 16 participants and nine facial positions. The $\Delta E_{\mathrm{SCIELAB}}*$ value between the TSR and the HIS was 6.2 ± 3.3, the between SPM and the HIS was 10.0 ± 4.3, and between the minHIS data and the HIS was 9.5 ± 4.9, with values representing the average and the standard error estimated across the 16 participants and nine facial positions.

Such results indicate that the differences presented in Table III were extendable to the estimation of the differences when using the GOF and the ΔE*_SCIELAB_ reinforcing those estimations of local and global color variations could only be estimated when using hyperspectral images with high spatial information. Each pixel that composes these hyperspectral images of faces had a visual angle of 1′ (0.017°), approximately, when a distance of observation of 1 m is considered, corresponding to the limits of the visual acuity of the human eye (where a minimum visual angle of 1′ of resolution was required for a 20/20 visual acuity).^112–114^

When comparing the HIS's results across different acquisition methodologies, the reflectance spectrum pattern agrees with previous studies,^27,28,53^ as for the found intra-subject spectral variability.^46^ The HIS and the TSR methods followed similar geometries of illumination and acquisition, providing closer comparable reflectance spectra and smaller color differences, as presented in Table III. The diffuse illumination and the 8° measuring angle of the SPM make the procedure quite different from the HIS and the TSR procedures, which correlates with higher RMSE values and higher color differences. The magnitude of the color differences estimated were considered as perceived by the human eye as they exceed the 2.2 chromatic discrimination threshold described elsewhere.^104^ Measures performed by noncontact devices may also be influenced by varying geometries of the illumination, shadows, and multiple light reflections by nonflat skin surfaces, like the nose and cheeks, which do not occur when using a contact instrument. Despite these sources of error, data presented here were acquired while attempting to minimize such sources of error by compensating acquired data for the illumination spatial variations and by attempting uniformity on the illumination by placing it distant from the face, centered to the face, and keeping it at the same position during all acquisitions regarding the participants’ position of the face. These possible influences of the illumination in the acquisition of the reflectance data, although possible, were then minimized and do not exist when considering the analysis of the variation of the reflectance data across a single area (or position) of the face. Also, contact devices may cause a color shift by blanching the skin when measuring the skin spectrum, due to the pressure of the instrument against the skin.^93,115^ The illumination/measurement geometry found in SPM is constant across all measurements which may account for less variability in the intensity of the reflectance spectra as presented in Figure 8c. Nevertheless, noncontact devices tend to represent closely the spectral reflectance distribution of nonuniform surfaces, as perceived in real environments, as conditions of viewing and acquisition are comparable.

The local spectral variations found in the hyperspectral images of Group 1 and Group 2 are independent of the number of images of human faces available in each group. The fewer images available in Group 2 may limit the direct comparison of the data between groups but demonstrate that the local spectral variations are consistently found for each group.

Due to the exposure time needed to acquire a single take with the HIS, in particular exposure times associated with lower wavelengths, facial movements could occur creating small artifacts that only affected small areas of the entire images. As an image of the face was acquired at each wavelength, these small facial movements could be computationally corrected by means of image registration or spectral reflectance rebuild.^48,53,108,109^ Nevertheless, presenting the data without correction provides a better ground truth, which does not limit future data analysis and processing. So, for the same skin sample, slight differences in the spectral measurements of the different devices should be expected.^22,96^ Yet, the repeatability and reproducibility of the hyperspectral imaging technique have been demonstrated previously,^57^ with the added advantage of providing spatial information of the entire face simultaneously and enhancing the visualization of some skin features by controlling spectral wavelength regulation.

## Conclusion

The skin of 29 human faces was imaged and the spectral reflectance was estimated for each pixel of the hyperspectral images. The HIS data acquired were ascertained with other measuring devices with different illumination/measuring geometries. It was found that local variations of the spectral profile of the human skin cannot be overlooked and assumed to be represented by the local average of the spectral reflectance of average chromaticity coordinates. Care must be taken when considering different facial locations, local variations, and inter-subject variations, independent of the type of skin considered.

## Supplemental Material

sj-docx-1-asp-10.1177_00037028241279323 - Supplemental material for Hyperspectral Imaging Database of Human Facial Skin Supplemental material, sj-docx-1-asp-10.1177_00037028241279323 for Hyperspectral Imaging Database of Human Facial Skin by Andreia E. Gomes, Sérgio M. C. Nascimento and João M. M. Linhares in Applied Spectroscopy
