# Supplementary material for: Hyperspectral Imaging Database of Human Facial Skin
Source: Appl Spectrosc. 2024 Sep 24;79(2):328–44. doi: 10.1177/00037028241279323 (PMC11823275; doi:10.1177/00037028241279323)
Supplement: sj-docx-1-asp-10.1177_00037028241279323 - Supplemental material for Hyperspectral Imaging Database of Human Facial Skin [file sj-docx-1-asp-10.1177_00037028241279323.docx]

**SUPPLEMENTAL MATERIAL**

**Hyperspectral Imaging Database of Human Facial Skin**

Andreia E. Gomes*, Sérgio M. C. Nascimento, João M. M. Linhares

Physics Center of Minho and Porto Universities (CF-UM-UP), University of Minho, 4710-057 Braga, Portugal

*Corresponding author email: jlinhares@fisica.uminho.pt

This chapter contains supplementary images intended to provide further visual context and support for the presented study. These images offer additional insights and clarity about the images of the faces.

**Materials and Experimental Methods**

*Participants*

Figure S1 represents examples of the types of faces assigned to each skin color group defined in Figure 1a; the face on the left was retrieved from Group 1, and the face from the right was retrieved from Group 2.


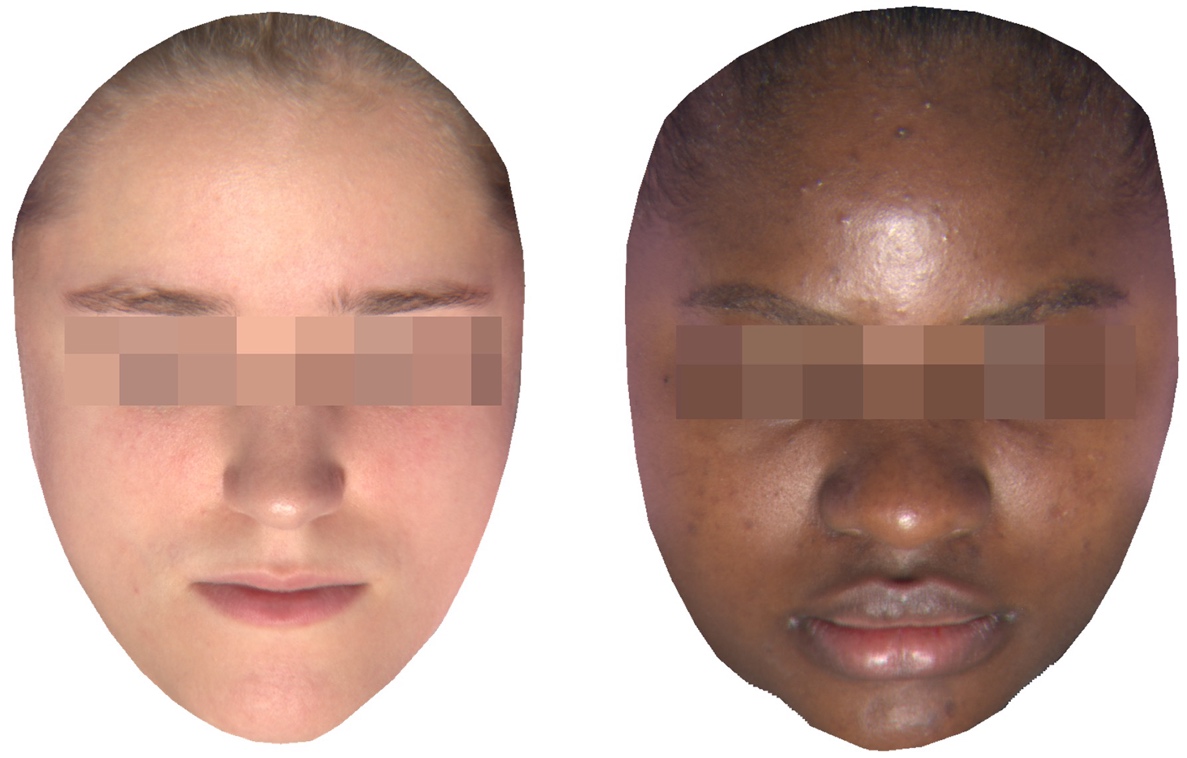


**Figure S1.** Examples of faces classified as Group 1 (left face) and Group 2 (right face). Images were redacted for identity protection.

*Hyperspectral Imaging Measurements*

Figure S2 represents local image artifacts caused by small eyelid or lip movements.


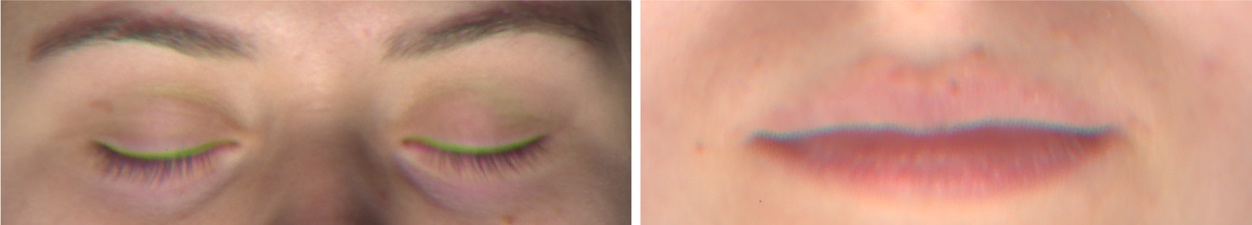


**Figure S2.** Small facial movements found in the hyperspectral images, only visible after image processing. The movement was visually identified by artificially colored areas, such as the green color around the base of the eyelashes (left image) and the bluish line between the lips (right image).

Figure S3 represents the data acquired using the HIS, for a set of selected individual wavelengths. The first image was rendered for color visualization purposes from the reflectance data assuming the CIE D65 illuminant and the CIE 2006 10° cone-fundamental-based colorimetric observer. Other images are grayscale images, with the minimum digital value of the image set to black and the maximum to white.


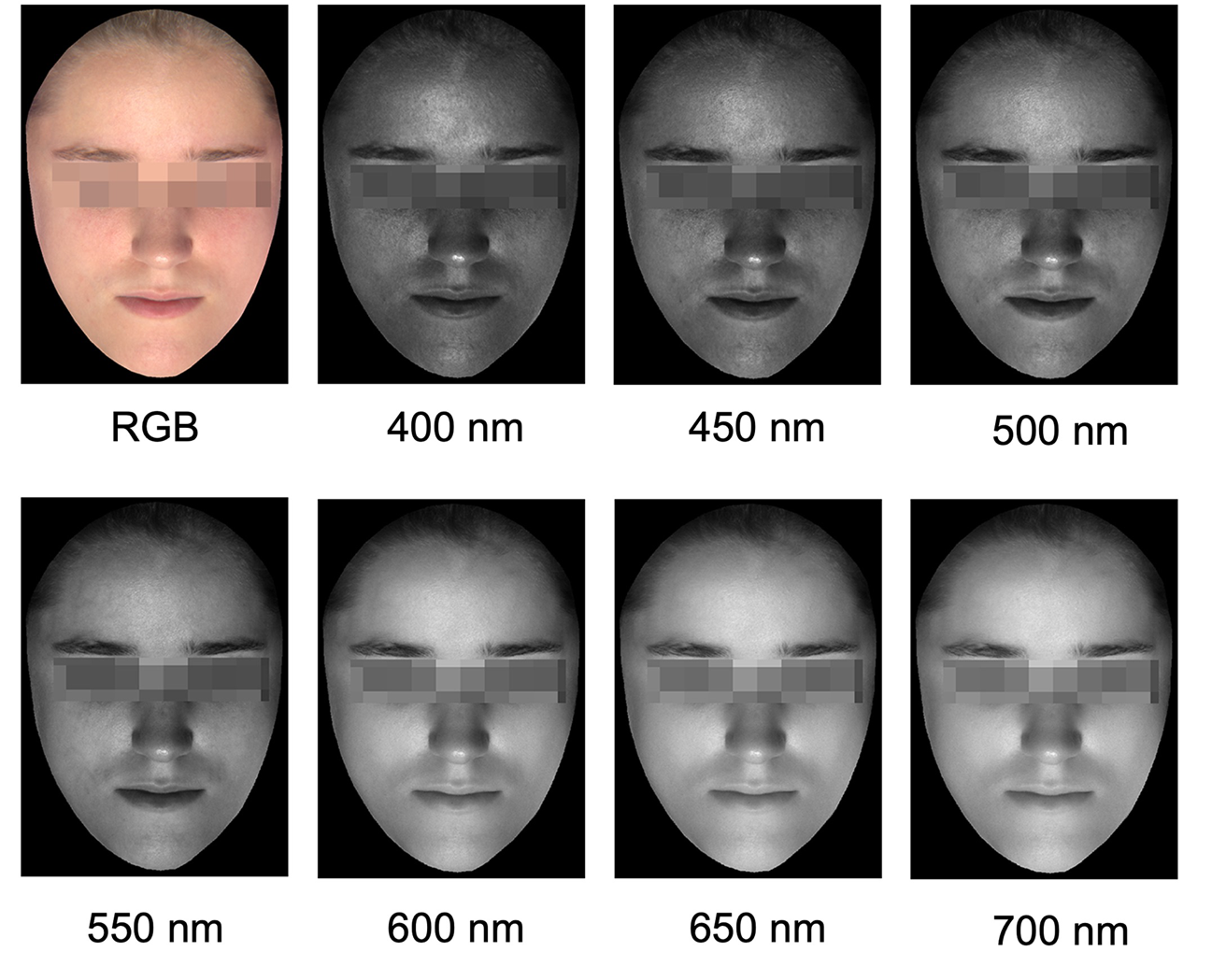


**Figure S3.** Images of the data acquired using the HIS for a sample of selected wavelengths. The first image is a colored image rendered from the spectral information, assuming the CIE D65 illuminant and the CIE 2006 10° cone-fundamental-based colorimetric observer. The remaining images are grayscale images, with the minimum digital value of the image set to black and the maximum to white. Images were redacted for identity protection.

**Results**

*Spectral and Colorimetric Analysis of the Hyperspectral Images*

Figure S4 represents the averaged spectral reflectance (black line) and correspondent standard deviation (gray shade) across Group 1 (Figure S4a) and Group 2 (Figure S4b) separately to better highlight the differences between areas of measurement. Colored lines represent the average for each one of the nine facial positions, from position 1 (P1) to 9 (P9), following the same color coding as represented in Figure 1a.


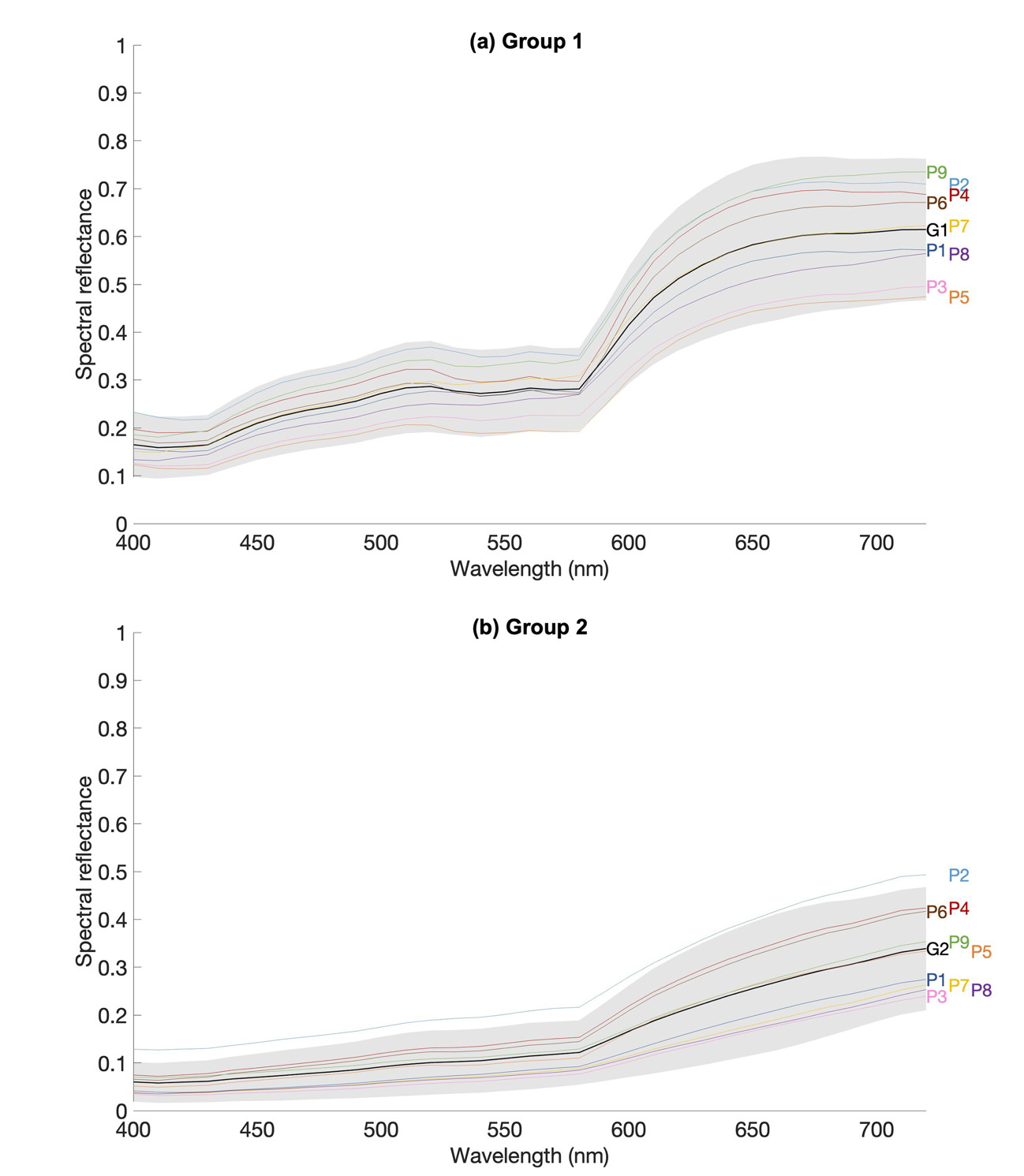


**Figure S4.** Average spectral reflectance (black line) and standard deviation (gray shade) across participants from the HIS image database of the 29 faces divided into (a) Group 1 (G1) and (b) Group 2 (G2). Colored lines represent the average spectral reflectance for each facial position (from P1 to P9), color-coded to the colors and areas represented in Figure 1a.

**Spectral and Colorimetric Analysis of the Hyperspectral Images**

*Results*

Figure S5 represents CIELAB chromaticity coordinates segmented for each individual facial position for Group 1 (G1, red dots) and Group 2 (G2, blue dots), with the correspondent projections in CIE (*a**, *b**) as light gray and dark gray, respectively. Each chromaticity coordinate represents the color of a single pixel.


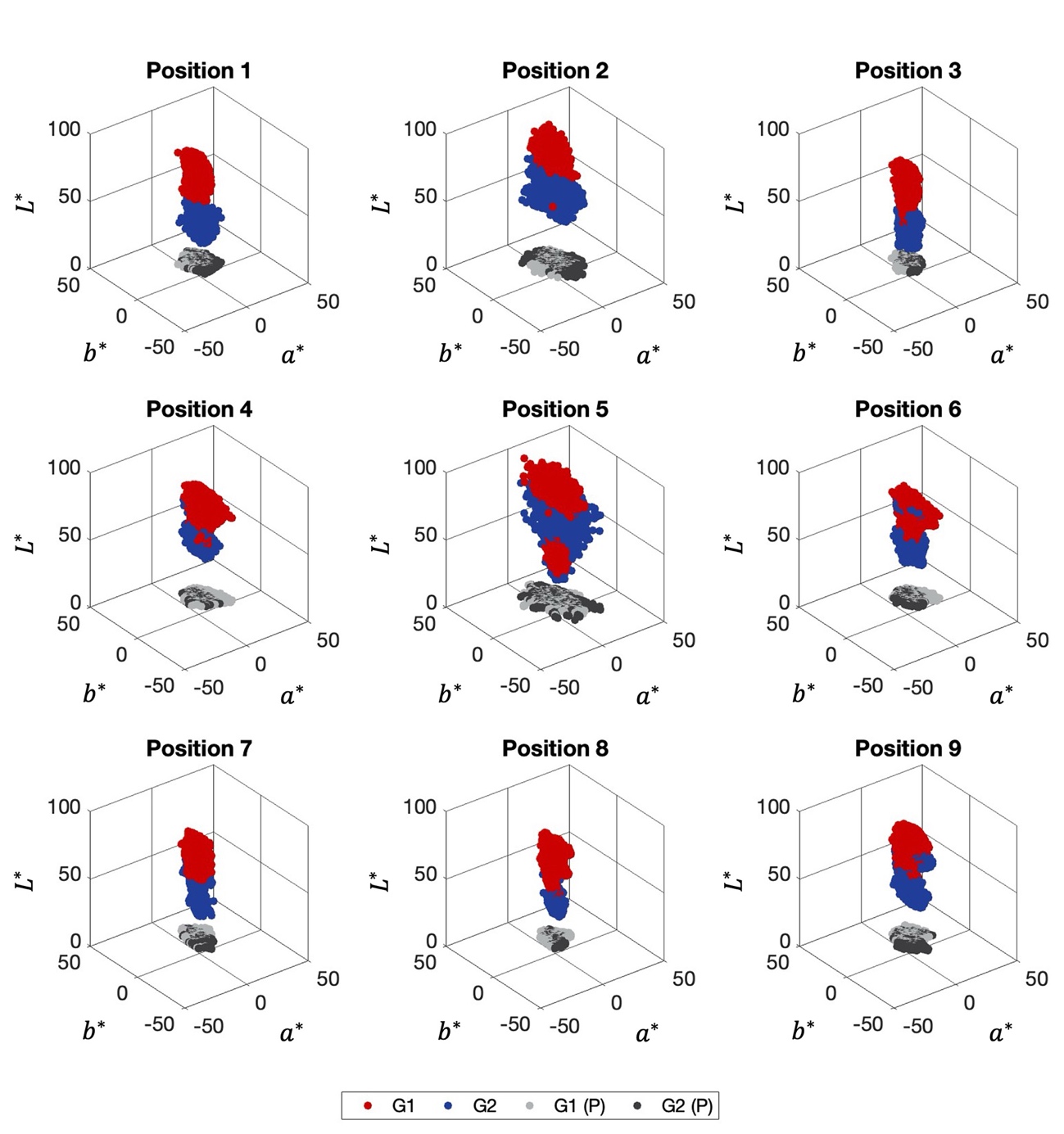


**Figure S5.** CIELAB color volume of the chromaticity coordinates for each of the nine facial positions, labeled from position 1 to position 9, for Group 1 (G1, in red) and Group 2 (G2, in blue) faces from all the images of the database. Gray areas represent the CIE (*a**, *b**) projections of the CIELAB color volume, as light gray for G1 and dark gray for G2. Position 1 (central forehead) and Position 2 (nose) present the higher color volume across all nine facial positions.
